# Mastermind Mutations Generate a Unique Constellation of Midline Cells within the *Drosophila* CNS

**DOI:** 10.1371/journal.pone.0026197

**Published:** 2011-10-27

**Authors:** Yi Zhang, Randi Wheatley, Eric Fulkerson, Amanda Tapp, Patricia A. Estes

**Affiliations:** Department of Genetics, North Carolina State University, Raleigh, North Carolina, United States of America; Columbia University, United States of America

## Abstract

**Background:**

The *Notch* pathway functions repeatedly during the development of the central nervous system in metazoan organisms to control cell fate and regulate cell proliferation and asymmetric cell divisions. Within the *Drosophila* midline cell lineage, which bisects the two symmetrical halves of the central nervous system, *Notch* is required for initial cell specification and subsequent differentiation of many midline lineages.

**Methodology/Principal Findings:**

Here, we provide the first description of the role of the *Notch* co-factor, *mastermind*, in the central nervous system midline of *Drosophila*. Overall, zygotic *mastermind* mutations cause an increase in midline cell number and decrease in midline cell diversity. Compared to mutations in other components of the *Notch* signaling pathway, such as *Notch* itself and *Delta*, zygotic mutations in *mastermind* cause the production of a unique constellation of midline cell types. The major difference is that midline glia form normally in zygotic *mastermind* mutants, but not in *Notch* and *Delta* mutants. Moreover, during late embryogenesis, extra anterior midline glia survive in zygotic *mastermind* mutants compared to wild type embryos.

**Conclusions/Significance:**

This is an example of a mutation in a signaling pathway cofactor producing a distinct central nervous system phenotype compared to mutations in major components of the pathway.

## Introduction

The central nervous system (CNS) of metazoan organisms consists of many different types of neurons and glia generated through the combinatorial action of intrinsic transcription factors and extrinsic signaling inputs from neighboring cells [1]–[3]. During CNS development and in a number of developmental contexts, the *Notch* pathway functions as a prominent signaling system providing positional input between cells in direct contact with one another [4], [5]. Previously, several roles for *Notch* during the development of specific cell lineages within the CNS midline of *Drosophila melanogaster* embryos have been described [6]. Here, we characterize functions of the co-activator, *mastermind* (*mam*) during the development of midline lineages.

One of the most surprising findings from comparative developmental biology is the extensive conservation of signaling pathways both within multiple tissues of a given organism as well as within the same tissue across diverse organisms. The *Notch* signaling pathway is a salient example and is used repeatedly to construct tissues during development and maintain homeostasis in adults [4], [7]–[9]. *Notch* signaling occurs between contacting cells when the Notch protein, a transmembrane receptor on the surface of one cell, binds one of its ligands, Delta (Dl) or Serrate/Jagged, on an adjacent cell. After binding one of these ligands, the Notch receptor is cleaved and its intracellular domain (*NICD*) transported to the nucleus where it interacts with the DNA-binding protein CSL (CBF1 in mammals, Suppressor of hairless (Su(H)) in *Drosophila*, and LAG-1 in *C. elegans*; hereafter referred to as Su(H); [10]). In cells devoid of *Notch* signaling, Su(H) functions as a repressor; whereas, in cells containing activated Notch, the *NICD* binds to both Su(H) and the co-activator Mam, resulting in a complex that activates transcription of target genes [11]–[14]. A striking example of the pleiotropic effects of *Notch* on a cell lineage can be found during CNS midline cell development in fruit flies [6]. In that study, *Dl* mutants were used to show that *Notch* promotes formation of midline glia and several midline neurons, while inhibiting the formation of other midline neurons.

**Figure 1:**
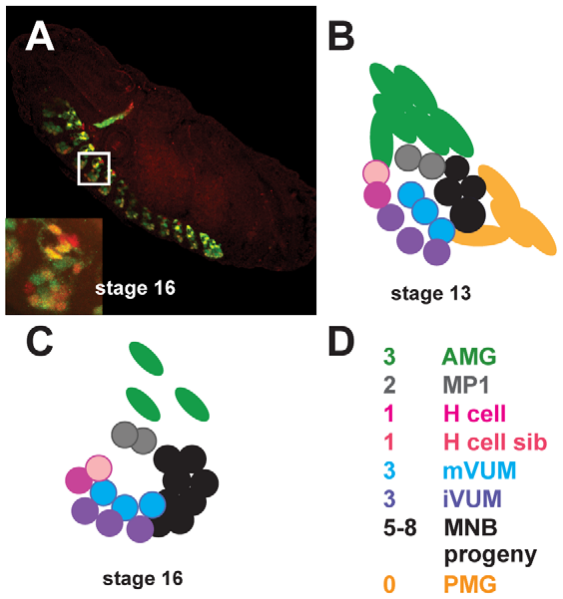
Overview of CNS midline cell development. (A) Confocal image of a stage 16 *Drosophila* embryo labeled with an anti-*sim* (red) and anti-*GFP* (green) antibody. The embryo contains a reporter gene that expresses *GFP* in all midline cells. A single segment of the CNS is indicated in the white box in A and shown in the inset located in the lower, left corner. Lateral views are shown; anterior is toward the top, left corner. (B–D) *Drosophila* midline cells within a single segment at (B) mid embryogenesis (stage 13) and (C) late embryogenesis (stage 16) are shown in lateral views, adapted from Wheeler et al., 2006 [31]. Each color corresponds to a particular midline cell type as listed (D; see text for a description of the cell types).

The CNS is located on the ventral side of the *Drosophila* embryo and consists of a repeated unit found within all thoracic and abdominal segments. Midline cells of *Drosophila* are located in the center of the embryonic CNS (Figure 1A) and they signal to and organize axons in a manner analogous to floor plate cells within the spinal cord of vertebrates, using similar signaling molecules [15], [16]. Because of its simplicity, the fly midline is used to study axon guidance as well as transcription factors and signaling pathways involved in nervous system development [17]–[19]. Previous studies indicate the initial specification of *Drosophila* midline cells depends on expression of *single-minded* (*sim*), the master regulator of this lineage [20]–[23]. Activation of *sim* in the cells that will give rise to the midline is directly controlled by dorsal/ventral patterning genes such as Dorsal, Twist and Snail, together with *Notch* signaling [24]–[26]. In subsequent stages (8–9), segment polarity genes such as *engrailed (en)*, *wingless* and *hedgehog* determine midline cell fates by separating the midline progenitor cells into anterior and posterior compartments [18], [27]. By the end of embryogenesis, the mature *Drosophila* midline consists of a small number of glia and neurons per segment (Figure 1A, C and D): approximately 3 anterior midline glial cells (AMG), 2 midline precursor 1 (MP1) neurons, 2 MP3 interneurons (the H cell and H cell sib), 3 ventral unpaired median interneurons (iVUMs), 3 ventral unpaired median motorneurons (mVUMs), and approximately 5–8 interneurons and motorneurons derived from the median neuroblast (MNB) [17], [28], [29]. Posterior midline glia arise transiently, but die by the end of embryogenesis [30], [31]. In summary, midline cells provide a tractable system for understanding how CNS neurons and glia are generated during embryogenesis.

Here, we provide the first study of *mam* functions in the various CNS midline lineages of *Drosophila*. The results indicate that both anterior and posterior midline glia (AMG and PMG) appear to form normally in *mam* mutant embryos, in contrast to midline glia in *Notch* and *Dl* mutants, which are completely absent. The presence of midline glia in *mam* mutants allows us to follow their development in late embryogenesis, when zygotic *mam* mutants cause an increase in the number of midline glia that survive in the mature CNS. In addition, *mam* and *Notch* mutants differ in the composition of MP1 neurons, whereas the other midline neural phenotypes observed in *mam* mutants are also observed in *Notch* and *Dl* mutants [6]. Further comparisons of *Notch* and *mam* mutants indicate that differences in the expression of the midline gene, *sim*, contribute to the observed difference in midline phenotypes. Taken together, the results demonstrate that zygotic mutations in the *mam* co-factor result in a midline cellular composition distinct from zygotic *Notch* mutations.

## Results

### *Mam* was identified in a screen for genes that function in midline development

**Figure 2:**
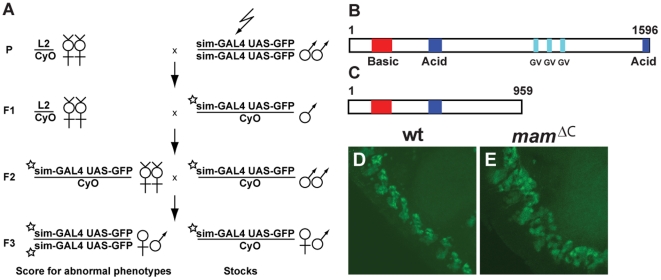
Midline cell development is disrupted in *mam^ΔC^* mutant embryos. (A) Genetic screen used to identify *mam* as a gene involved in midline cell development (see Materials and Methods for details of the screen). The jagged arrow represents the mutagen EMS fed to parental males and the star represents a resultant mutation on the second chromosome. (B) Mam protein contains one N-terminal basic cluster (amino acids 127–256), 2 acidic clusters (amino acids 466–539 and 1559–1592), and 3 runs of glycine-valine (GV) residues (amino acids 987–1000; 1094–1110; and 1236–1257). (C) The *mam^ΔC^* mutation creates a premature stop codon that results in a truncated protein ending at residue 959, eliminating the C-terminal acidic cluster and all 3 GV runs. Shown are confocal images of stage 16 (D) wild type and (E) *mam^ΔC^* mutant embryos containing the midline reporter combination *UAS-GFP sim-GAL4*. Lateral views are shown; anterior is toward the top, left corner.

To identify genes involved in *Drosophila* midline development, we used EMS to introduce mutations throughout the genome of the fly and then examined midline cells using a reporter gene combination that drives *GFP* expression in all midline cells (*UAS-GFP sim-GAL4*). In this way, *GFP* could be visualized and followed in live embryos during late embryonic and larval development; stages that are difficult to examine using routine immunostaining techniques. 1037 lines carrying lethal mutations on the second chromosome were established and embryos from each line were collected and examined for midline cell defects (Figure 2A). Of the 1037 lethal lines screened, 21 showed midline defects based on the *UAS-GFP sim-GAL4* reporter. These mutations were mapped within the genome using complementation; first with deficiency lines and then with fly lines containing mutations in single genes. In this report, we focus on one of the mutations that disrupted midline development and mapped to the *mam* locus [32]–[34]. Mam encodes the transcriptional co-activator of canonical *Notch* signaling [12] and is a glutamine-rich nuclear protein with a predicted 1596 amino acid sequence [35]. The protein contains a highly conserved basic domain within the N-terminus that binds to both the *NICD* and Su(H); and 3 glycine-valine (GV) runs and 2 acidic clusters in the C-terminal region needed for 1) interactions with p300 and RNA polymerase and 2) stability of the NICD/Mam/Su(H) complex (Figure 2B; [32], [36]–[39]). Sequence analysis of the *mam^ΔC^* allele isolated in our screen predicts it encodes a truncated protein lacking both the C-terminal acid cluster and the GV runs (Figure 2C) and our phenotypic analysis indicates it behaves as a strong loss of function mutation (see below). The midline of *mam^ΔC^* mutant embryos was disorganized and less compact than the midline of wild type embryos during late embryonic stages (Figure 2D and E). Numerous studies have described *mam* functions in CNS development [40]–[42], yet its role in midline development has not been reported. This, the midline phenotype of *mam^ΔC^* mutant embryos and the previously characterized roles of *Notch* signaling during midline cell development, led us to investigate how various midline lineages were affected in *mam^ΔC^* mutant embryos.

### AMG and PMG are present in *mam^ΔC^*, but not *N^55e11^* mutant embryos

**Figure 3:**
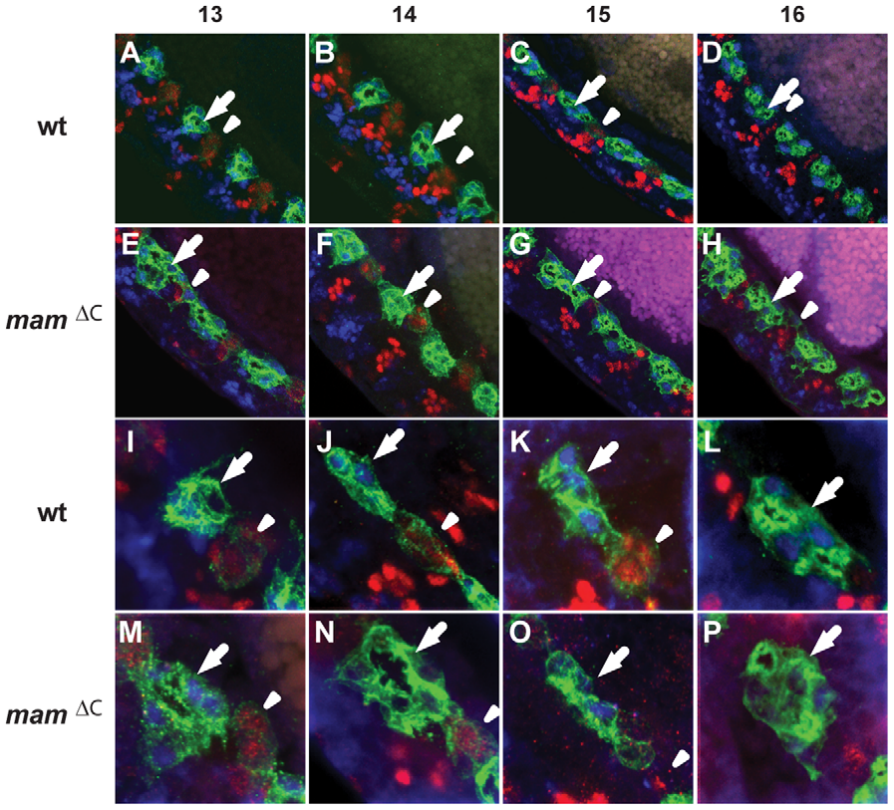
Additional AMG survive in *mam^ΔC^* mutant embryos compared to wild type embryos. Wrapper protein (green) is present in all midline glia and co-localization with Runt (blue; arrows) identifies AMG and with En (red; arrowheads) identifies PMG. 3–4 segments of (A–D) wild type and (E–H) *mam^ΔC^* mutant embryos are shown and higher magnification views of one segment within the CNS of (I–L) wild type and (M–P) *mam^ΔC^* mutant embryos are also shown. At stage 13, both (A and I) wild type embryos and (E and M) *mam^ΔC^* mutants have approximately 6 AMG and 4 PMG. During stages 14 and 15, AMG and PMG in both (B, C, J and K) wild type and (F, G, N and O) *mam^ΔC^* mutants diminish [44], [45]. By stage 16, the PMG are absent in both (D and L) wild type and (H and P) *mam^ΔC^* mutant embryos, whereas wild type embryos contain 3 AMG and *mam^ΔC^* mutants contain about 5 (Table 1). Images are projections of multiple focal planes and cells were counted using stacks of all focal planes.

**Figure 4:**
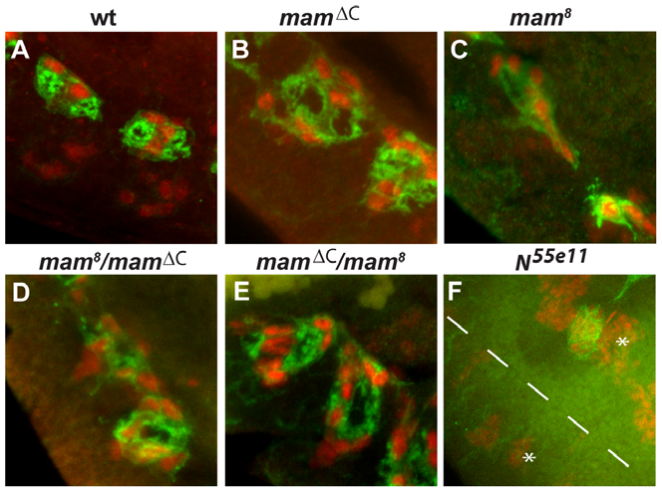
Comparison of AMG in *mam^ΔC^* and *mam^8^* mutant embryos. AMG are present within (B) *mam^ΔC^* and (C) *mam^8^* homozygous mutant embryos as well as (D and E) *mam^ΔC^*/*mam^8^* transheterozygous embryos. AMG in each mutant combination are disorganized compared to (A) wild type embryos. In contrast, midline glia cannot be detected in (F) *N^55e11^* mutant embryos during late embryogenesis. Transheterozygous embryos were generated by crossing either (D) *mam^8^* heterozygous females to *mam^ΔC^* heterozygous males or (E) *mam^ΔC^* heterozygous females to *mam^8^* heterozygous males. Co-localization of *sim* (red) and *wrapper* (green) was used to compare the phenotype of AMG in different genetic backgrounds during late embryogenesis (stage 16). The stars indicate muscle precursors expressing *sim* and the dotted line indicates the ventral midline of the *N^55e11^* mutant embryo.

Previous lineage analysis suggested midline glial precursors undergo multiple divisions to give rise to 2 populations of midline glia at late stages [6], [30], [43]. At stage 13, each segment contains about 6 AMG derived from the anterior compartment of the segment that express *runt* but not *en*; and 4 PMG cells, derived from the posterior compartment that express *en* but not *runt* (Figure 1B). Later, at stage 16, only 3 AMG survive to enwrap the axon commissures, while all of the PMG and remaining AMG are depleted by apoptosis [43]–[45]. Both AMG and PMG are missing in *Dl^3^* mutants, suggesting the *Notch* pathway is required for development of both glial lineages [6]. To examine midline glial development in *mam^ΔC^* mutant embryos, we monitored Wrapper, an immunoglobulin protein required for midline glial survival, and expressed almost exclusively in the midline glia, at a high level in AMG and a lower level in PMG [43], [46]. The development of AMG can be followed using the co-localization of Wrapper and Runt, while the PMG can be identified using co-localization of Wrapper and En (Figure 3A–P). During mid and late embryogenesis, Wrapper protein was never detected in the midline of *N^55e11^* homozygous embryos, a null allele of *Notch* (Figure 4F and S1A), but present at high levels in the AMG and at lower levels in the PMG of wild type and *mam^ΔC^* mutant embryos (Figure 3A–P). At stage 13 (mid embryogenesis), both wild type and *mam^ΔC^* mutants contained 6 AMG per segment (Figure 3A and I; Table 1). Wild type embryos contained 4 PMG, whereas *mam^ΔC^* mutants contained about 3 per segment (P = 0.0001; Figure 3A and I; Table S2). By stage 16 (late embryogenesis), wild type embryos contained just 3 AMG (Figure 3D and L; Table 1), whereas *mam^ΔC^* mutants contained approximately 5 AMG (P = 0.0001; Table 1; Figure 3H and P). The PMG were not detectable at stage 16 in wild type or *mam^ΔC^* mutant embryos (Table S2). In addition, midline segmental compartments were less clearly defined and in many cases, glial processes extended into the posterior compartment in *mam^ΔC^* mutants (Figure 3E–H and M–P) compared to wild type embryos (Figure 3A–D and I–L). These results show that *mam^ΔC^* mutants, in marked contrast to *N^55e11^* mutants, contained AMG and PMG and that additional AMG survived during late embryogenesis in *mam^ΔC^* mutants compared to wild type embryos.

**Table 1:** Comparison of AMG in wild type, *mam^ΔC^* and *N^55e11^* mutant embryos and embryos overexpressing *Notch* and *EGFR* signaling components during mid and late embryogenesis.

Genotype	stage 13			stage 16		
**wild type**	6.3±0.27	a	(18)	2.9±0.16	a	(14)
***mam^ΔC^***	6.4±0.41	a	(9)	4.7±0.40	bc	(10)
***N^55e11^***	0.0±0.00	b	(11)	0.0±0.00	e	(9)
**UAS-NICD**	16±0.43	c	(15)	6.1±0.27	bd	(17)
**UAS-Su(H)**	16±0.37	c	(11)	6.7±0.19	d	(12)
**UAS-mam**	6.6±0.29	a	(21)	2.2±0.94	a	(15)
**UAS-sspi**	9.5±0.61	d	(11)	4.9±0.22	bc	(14)
***mam^ΔC^*** ** UAS-sspi**	7.7±1.01	ad	(9)	4.5±0.62	c	(13)

The number of AMG found in a single CNS segment of wild type, *mam^ΔC^* and *N^55e11^* mutants and embryos overexpressing the *NICD* (*UAS-NICD sim-GAL4*), Su(H) (*UAS-Su(H) sim-GAL4*), *mam* (*UAS-mam sim-GAL4*) or *spi* (*UAS-sspi sim-GAL4*) in the midline and embryos overexpressing *spi* in the midline of *mam* mutants (*mam^ΔC^ UAS-sspi/mam^ΔC^ sim-GAL4*) at stages 13 and 16 is shown. Results are shown as means ± SEM and the sample size is indicated in parentheses. Stage 13 ANOVA: *F*_8,110_ = 140.18, *P* = 0.0001 and stage 16 ANOVA: *F*_7,90_ = 147.99, *P* = 0.0001. Within a column, treatments with different letters are significantly different (Tukey-Kramer HSD, *P*<0.05).

### Embryos containing *mam* deletions also contain AMG

Because the *mam^ΔC^* mutation introduces a premature stop codon, the N-terminus of the resultant protein is still present and may be able to interact with the NICD and Su(H) to form an activation complex [47]. If so, the *mam^ΔC^* allele may retain some function and act as either a hypomorph or dominant negative allele. To test this, we examined midline phenotypes of embryos homozygous for a characterized point mutation in *mam* (*mam^8^*; [34], [48]) as well as several chromosome deletions that lack all or part of the *mam* gene: *Df(2R)BSC383*, *Df(2R)50C-38*, and *Df(2R)BSC18* (Figure S2A). Midline glia were clearly present in homozygous *mam^ΔC^* (Figure 4B; see also Figure 3) and *mam^8^* mutants (Figure 4C), as well as *mam^8^*/*mam^ΔC^* transheterozygotes (Figure 4D and E). The N-terminal region of the *mam* protein is absent in *Df(2R)BSC383* and the entire *mam* gene is deleted in *Df(2R)BSC18* and *Df(2R)50C-38* (Figure S2A). In homozygous *mam* deficiency mutants, Wrapper protein was also clearly detectable (Figure S1C–E), indicating the presence of AMG. The CNS midline in homozygous *Df(2R)50C-38* and *Df(2R)BSC383* embryos appeared more disorganized than in homozygous *Df(2R)BSC18* embryos (Figure S1B–E), possibly due to additional genes missing in these larger deletions. The results indicate that the midline glia were present in all homozygous point and deficiency embryos tested, similar to results obtained with *mam^ΔC^* mutants (Figure 3E–H and M–P), but different from those obtained with *N^55e11^* mutants which lack midline glia (Figure 4F and S1). These results suggest that the *mam^ΔC^* mutant behaves as a strong loss of function allele and that midline glia do form in embryos completely lacking zygotic *mam* activity.

### Additional AMG survive in *mam* mutant embryos

**Figure 5:**
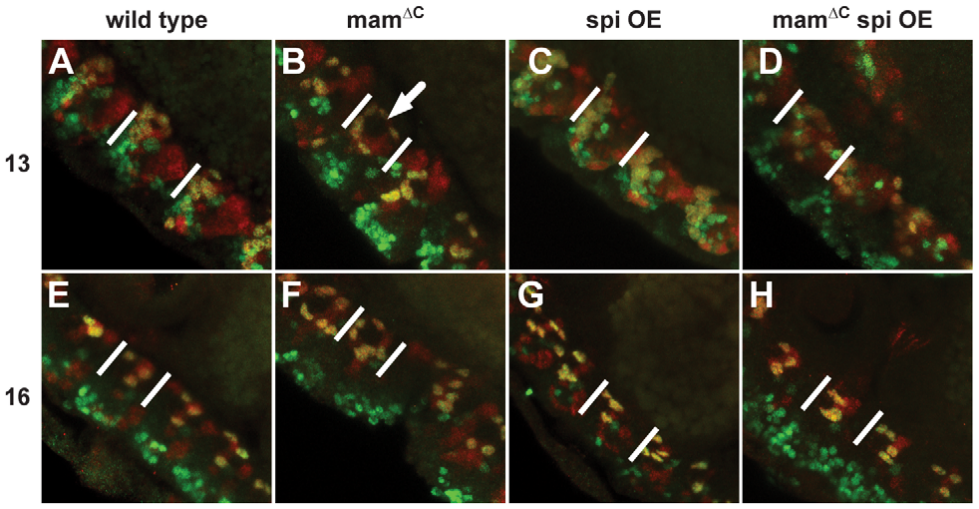
During late embryogenesis, the number of AMG that survived was similar within both *mam^ΔC^* mutant embryos and embryos overexpressing Spi. Co-localization of *sim* (red) and *runt* (green) was used to compare the number of AMG in different genetic backgrounds during mid and late embryogenesis. At mid embryogenesis, (A) wild type embryos contain a compact cluster of approximately 6 AMG and (B) *mam^ΔC^* mutant embryos have approximately the same number of AMG, but are less compact (arrow). During mid embryogenesis, (C) overexpression of Spi causes an increase in AMG to approximately 9 AMG, while (D) overexpression of Spi in a *mam^ΔC^* background resulted in a number of AMG indistinguishable from both wild type and Spi overexpression embryos. During late embryogenesis, (E) 3 AMG are present in wild type embryos, while (F) *mam^ΔC^* mutant embryos, (G) embryos overexpressing *spi* and (H) embryos overexpressing *spi* in a *mam^ΔC^* mutant background, all contain about 5 AMG. Statistical comparisons of AMG cell counts are shown in Table 1. Lateral views of (A–D) stage 13 and (E–H) 16 embryos are shown and white lines indicate individual CNS midline segments.

**Figure 6:**
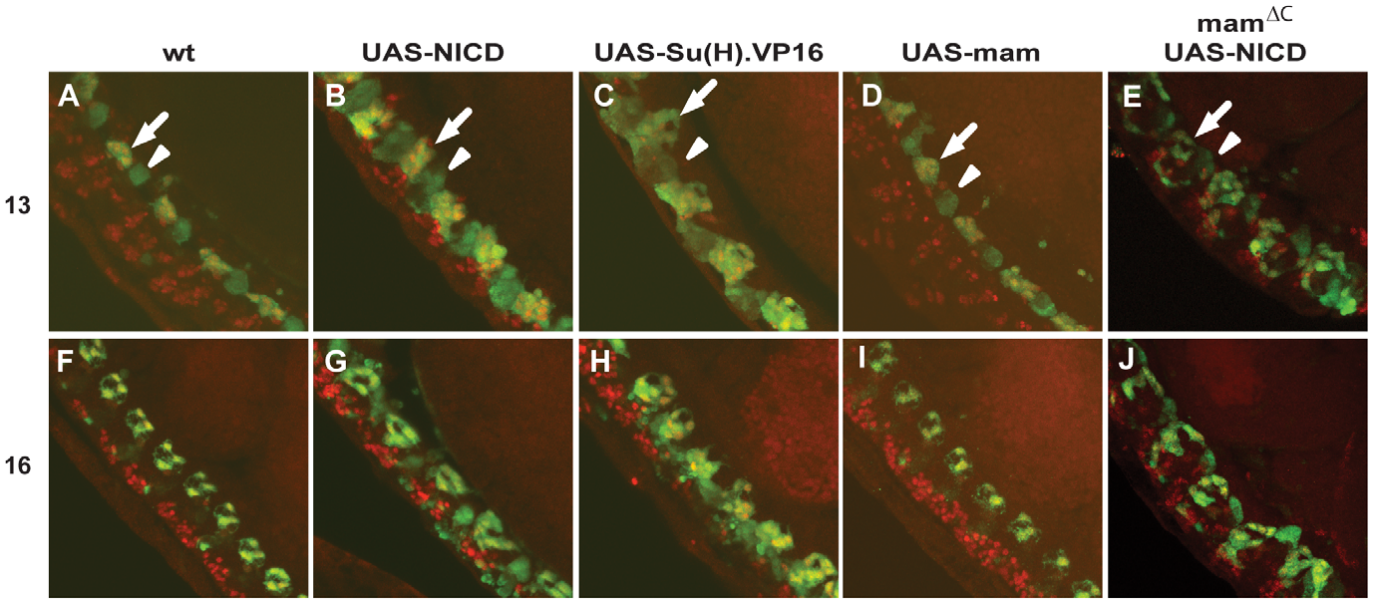
Overexpression of either the *NICD* or *Su(H)*.*VP16*, but not *mam*, causes an increase in the number of AMG. (A–J) Analysis of a *wrapper*:*GFP* reporter gene indicates *GFP* expression is higher in the AMG (arrow) than in the PMG (arrowhead). During mid embryogenesis, the number of AMG increased when (B) the *NICD* in a wild type background or (E) the *NICD* in a *mam^ΔC^* mutant background, or (C) *Su(H)*.*VP16* was overexpressed in the midline, whereas AMG number was unchanged compared to (A) wild type when (D) *mam* was overexpressed in the midline. During late embryogenesis, more AMG survived in embryos overexpressing (G) the *NICD* in a wild type background or (J) the *NICD* in a *mam^ΔC^* mutant background or (H) *Su(H)*.*VP16* in the midline, whereas embryos overexpressing (I) *mam* in the midline contained about the same number of AMG as (F) wild type embryos. Shown are whole-mount (A and F) wild type, (B and G) *UAS-NICD sim-GAL4*, (C and H) *UAS-Su(H)*.*VP16 sim-GAL4* (D and I) *UAS-mam sim-GAL4* and (E and J) *UAS-NICD sim-GAL4* in a *mam^ΔC^* mutant background. Statistical comparisons of AMG cell counts are shown in Table 1. Shown are lateral views of embryos labeled with anti-*GFP* (green) and anti-Runt (red) antibodies; anterior is toward the top, left corner.

**Figure 7:**
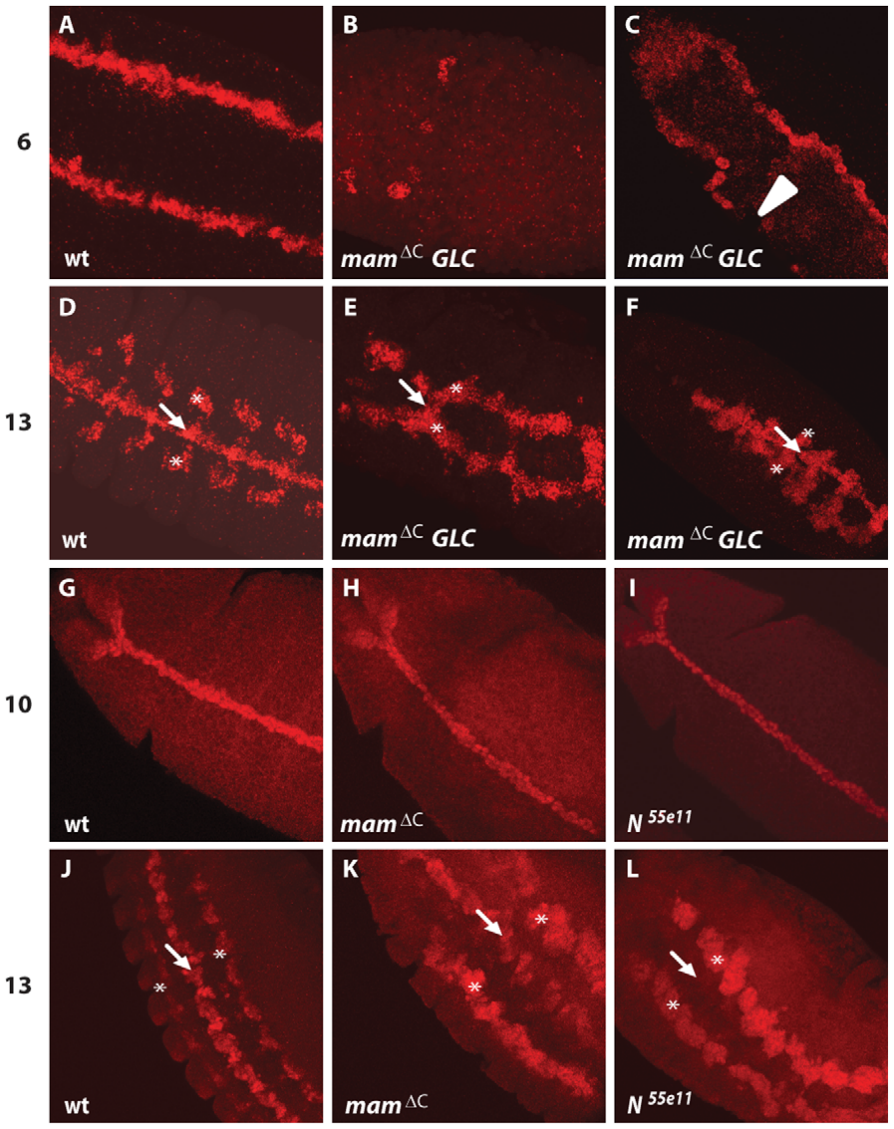
Proper initiation of *sim* expression requires maternal *mam* function, whereas the maintenance of *sim* expression requires zygotic *Notch* but not *mam*. (A–F) *Sim* expression was examined in *mam^ΔC^* germline clones using fluorescent *in situ* hybridization. (A) In wild type blastoderm embryos, the mesectoderm consists of two rows of *sim* positive cells. (B and C) Embryos derived from *mam^ΔC^* homozygous mutant mothers exhibited a range of mutant phenotypes typified by embryos expressing very little *sim* (B) and embryos lacking *sim* expression in certain regions (arrowhead; C). (D) In wild type embryos, *sim* is strongly expressed in both midline (arrow) and muscle precursor cells (asterisks) at stage 13. (E and F) In some embryos derived from *mam^ΔC^* mutant mothers, midline expression of *sim* (arrow) was largely absent and muscle precursors were expanded (asterisk), as observed in *Notch* mutants [71]. (G–L) Sim expression in zygotic *mam^ΔC^* and *N^55e11^* mutants was analyzed using an anti-Sim specific antibody. (G–I) During stage 10 of embryogenesis, Sim expression appears normal in (H) *mam^ΔC^* and (I) *N^55e11^* mutants compared to (G) wild type. (J) At stage 13, Sim is expressed in the midline (arrow) and muscle precursor cells (asterisks) in wild type embryos. (K) In *mam^ΔC^* mutant embryos, Sim expression is slightly reduced in the midline (arrow) and muscle precursors appear expanded (asterisks). (L) In *N^55e11^* mutant embryos, Sim expression is absent in the midline (arrow), but present in muscle precursor cells (asterisks). Ventral or ventrolateral views are shown; anterior is toward the top, left corner.

**Figure 8:**
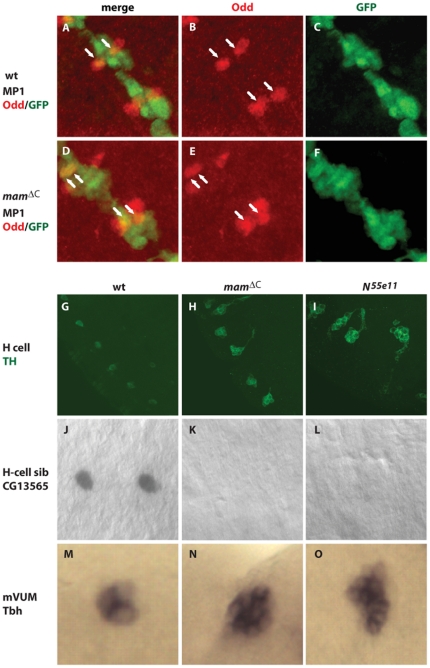
Mam functions in the canonical *Notch* pathway to control the development of midline neurons. Development of midline neural lineages was followed using specific markers. (A–C) Wild type embryos contain 2 MP1 neurons per segment (arrows), as do (D–F) *mam^ΔC^* mutant embryos, as monitored with (B and E) anti-Odd-skipped and (C and F) anti-*GFP* antibodies, together with the *UAS-GFP sim-GAL4* reporter. (A and D) In this experiment, MP1 neurons express both Odd and *GFP*, whereas MP2 neurons express only Odd. (G) Wild type embryos contain one TH positive H cell in each segment, while (H) *mam^ΔC^* mutant embryos contain 6 and (I) *N^55e11^* mutant embryos contain 10. (J) One H cell sib per segment was present in wild type embryos, while the H cell sib was absent in (K) *mam^ΔC^* and (L) *N^55e11^* mutant embryos, as monitored with the marker *CG13565*. (J–L) Two segments within stage 15 embryos are shown. (M) Three *Tbh* positive mVUM neurons were present within each segment of wild type embryos, whereas (N) *mam^ΔC^* and (O) *N^55e11^* mutant embryos each contained about 11 per segment. (M–O) One segment of stage 13 embryos is shown. Gene expression was monitored using (A–I) specific antibodies or (J–O) *in situ* hybridization. (A–F) Ventral views with anterior toward the top left corner, (G–I) Lateral views with anterior toward the top, left corner or (J–O) ventral views with anterior on the left, are shown. Statistical comparisons of midline cell counts are shown in Table S1.

As described above, analysis of *mam* mutants indicated they contained additional AMG during late embryonic stages. To further investigate the AMG in *mam^ΔC^* mutant embryos, we investigated the interaction between *mam* and the *EGFR* signaling pathway, which is known to affect AMG survival. For these cells to survive, they must receive Spitz (Spi) from lateral CNS axons that cross the midline [44]. In AMG that die, the Head Involution Defective (HID) protein is active and stimulates apoptosis, whereas in surviving AMG, cell surface *EGFR* binds to Spi, leading to HID phosphorylation. Phosphorylated HID is inactive, and therefore, Spi-activated glia survive. Because *Notch* and *EGFR* signaling act antagonistically in many tissues [49]–[51], we wanted to determine their relationship in AMG. However, this is not possible in *Notch* mutants because they lack midline glia. Instead, we investigated interactions between *mam* and *EGFR* in AMG by overexpressing the secreted form of Spi in the midline of *mam^ΔC^* mutant embryos. As described above, we found approximately 6 AMG per segment in both wild type and *mam^ΔC^* mutant embryos during mid embryogenesis, using the co-localization of Sim and Runt (Table 1; Figure 5A and B). Embryos overexpressing *spi* had a significant increase in AMG (P = 0.001; Table 1 and Figure 5C) to 8 per segment at stage 13. During this stage, embryos overexpressing *spi* in a *mam^ΔC^* mutant background could not be distinguished from embryos expressing *spi* in a wild type background or wild type embryos (Table 1 and Figure 5D). By late embryogenesis, the number of AMG in wild type embryos decreased to approximately 3 per segment as previously reported ([6]; Figure 5E). Interestingly, all 3 classes: 1) *mam^ΔC^* mutants, 2) embryos overexpressing *spi*, and 3) embryos overexpressing *spi* in a *mam^ΔC^* mutant background each had around 5 AMG per segment and each class was significantly different from wild type embryos (Table 1 and Figure 5F–H). This, together with the known neurogenic nature of *mam* mutations [42], suggested midline glia may be exposed to additional *spi* provided by the extra neurons generated in *mam^ΔC^* mutants. To investigate this, we compared the interaction of the midline glia with lateral axons in wild type and *mam^ΔC^* mutant embryos using Wrapper and the BP102 monoclonal antibody (Figure S1D–I). The results indicate that the additional AMG present in these embryos do enwrap lateral axons and have increased glial processes that stain with the *wrapper* antibody (see also Figure 3M–P). Moreover, the nerve cord does not retract normally in *mam^ΔC^* mutant embryos (data not shown), which may also be a consequence of extra neural tissue present in these embryos. These results suggest the greater number of neurons generated in *mam^ΔC^* mutant embryos may provide excess *spi* that allows additional AMG to survive.

### *Notch* activation expands expression of a Wrapper reporter

Results described above as well as previous studies [6] suggest *Notch* signaling promotes AMG and PMG development, which are completely absent in *N^55e11^* zygotic mutants. Because the AMG developed normally in *mam^ΔC^* zygotic mutants, we next compared the effect of overexpressing *mam* to the overexpression of other *Notch* signaling components. For these experiments, we examined both the presence of AMG using a Runt antibody, as well as the regulation of gene expression within AMG using a *wrapper* reporter gene. The reporter contains an 884 bp *wrapper* enhancer sufficient to drive expression of the *GFP* reporter gene in midline glia (Figure 6A and F; [52]). Expressing a constitutively active form of Su(H), *UAS*-*Su(H)*.*VP16* [53], in all midline cells using *sim*-*GAL4*, causes a three-fold increase of midline glial cells at the expense of midline neurons [6]. Expression of the *wrapper* transcriptional reporter was greatly expanded when either the *NICD* (Figure 6B) or *Su(H)*.*VP16* (Figure 6C) was overexpressed in the midline using the *sim-GAL4* driver. Co-localization with Runt indicated the expansion was due to the formation of additional AMG expressing the reporter compared to wild type embryos (Table 1; Figure 6A). Likewise, significantly more AMG survived until stage 16 in the *NICD* and *Su(H).VP16* overexpression embryos compared to wild type embryos (Table 1; Figure 6F, G and H) as previously reported [6]. Therefore, over activation of the *Notch* pathway in the midline led to an increase in the number of AMG as well as activation of the *wrapper* reporter in the additional cells.

In contrast, *UAS*-*mam sim*-*GAL4* embryos at both embryonic stages 13 and 16 appear normal and showed no increase in AMG at stage 16 (Figure 6D and I). Finally, embryos in which the *NICD* was overexpressed in the midline of *mam^ΔC^* mutant embryos also contained extra AMG (Figure 6J), similar to embryos overexpressing the *NICD* in a wild type background (Figure 6G). These results suggest AMG can form in the absence of zygotic *mam* function.

### AMG do not form in *mam^ΔC^* germline clones

Midline glia may form in zygotic *mam^ΔC^* mutant embryos because maternal *mam* transcripts are stable and produce sufficient Mam protein to function during *Notch* signaling when glia differentiate. To determine if AMG can form in embryos lacking maternal *mam* transcripts, we generated *mam^ΔC^* germline clones using the *FRT*, *hsFLP* system [54] and examined *wrapper* expression. Both *Notch* [55] and *mam* [32] are maternally deposited and germline clones of either gene exhibit a strong neurogenic phenotype [42]. We observed variable phenotypes in *mam^ΔC^* germline clones and many embryos had gross developmental defects. Most embryos did not express *wrapper*, although some did express this gene at low and variable levels and often in only limited regions of the embryo (Figure S1B and C). Embryos containing either one or no copies of *mam* had the same phenotypes, suggesting that it was the maternal and not zygotic *mam* activity that caused the reduction in *wrapper* expression.

Because zygotic *mam^ΔC^* mutants expressed *wrapper* at high levels, while *mam^ΔC^* germline clones did not, we compared midline development in embryos lacking either maternal or zygotic *mam* at earlier developmental stages. For these experiments, we examined *sim* expression, which is first activated at the blastoderm stage in the mesectoderm. Mesectodermal cells are located between the mesoderm and ectoderm on both sides of the embryo (Figure 7A) and *Notch* is needed in these cells for initial *sim* activation [22], [26], [56]. We determined if *mam* functions together with *Notch* to activate *sim* by examining *mam^ΔC^* germline clones. Wild type embryos express *sim* in the mesectoderm throughout the length of the embryo (Figure 7A) at the blastoderm stage. In contrast, most embryos derived from homozygous *mam^ΔC^* mutant mothers contained gaps in *sim* expression, and many embryos expressed *sim* in only a few cells (Figure 7B and C). The observed variation in *sim* expression is similar to that observed in embryos derived from *Notch* germline clones [26], [56]. As development progresses, the mesoderm invaginates at gastrulation and mesectodermal cells move toward and meet at the ventral midline. After this, *sim* was expressed at high levels in both midline and muscle precursors of wild type embryos (Figure 7D), whereas *sim* expression was low or undetectable in the midline, and expanded in muscle precursor cells of embryos derived from *mam^ΔC^* germline clones (Figure 7E and F). These results indicate that maternal *mam*, similar to maternal *Notch*, is required to activate *sim* during early *Drosophila* development.

### *sim* maintenance is disrupted in *N^55e11^*, but not *mam^ΔC^* zygotic mutants

Because germline clones of either *mam* or *Notch* lack *sim* expression early in development, midline cells do not develop [22] and the various midline lineages cannot be examined in these embryos. Therefore, to examine zygotic roles for *mam* and *Notch* on *sim* expression, we used our *mam^ΔC^* allele and the *N^55e11^* allele. We first determined if early *sim* activation was affected in *mam^ΔC^* zygotic mutants produced by wild type mothers (*mam^ΔC^* heterozygotes) and compared the results to zygotic *N^55e11^* mutants. *Sim* expression is normal until stage 10 in zygotic *mam^ΔC^* mutants (Figure 7H) and persists in subsequent stages, although at a reduced level (Figure 7K). *Sim* expression in the midline of *N^55e11^* mutant embryos also appeared normal at stage 10 (Figure 7I), but completely disappeared by stage 13 (Figure 7L). These results indicate that, unlike maternal mutations in *mam^ΔC^* and *N^55e11^*, zygotic mutations in these genes do not affect early *sim* expression prior to stage 10 and can therefore, be used to study their functions during subsequent stages of midline development. Moreover, the results indicate maternal *mam^ΔC^* and *N^55e11^* mutations have similar effects on *sim* expression during early development, whereas *sim* expression is maintained in zygotic *mam^ΔC^*, but not *N^55e11^* mutants during mid and late embryogenesis.

### The formation of certain midline neurons requires both *Notch* and *mam*

Next, we examined the effects of the *mam^ΔC^* mutation on the development of midline neurons. During embryonic stage 11, midline precursors (MPs) delaminate and divide to produce 6 neuronal subtypes [6], [28]. The MPs (1–6) are named based on their anteroposterior position within the segments of the CNS and each midline neural cell type (Figure 1B–D) expresses a unique gene combination that can be used to follow them during development [31]. We selected tractable markers for the various midline lineages to examine their fate in *mam^ΔC^* mutants.

We first examined the MP1 neural lineage, located within the anterior most region of each midline segment, using an Odd-skipped (Odd) antibody [31]. Odd labels 2 MP1 cells and 2 nearby MP2 cells in each CNS segment of wild type embryos (Figure 8A). To distinguish the MP1 and MP2 neurons, we utilized the *UAS-GFP sim-GAL4* reporter that labels MP1, but not MP2 neurons. *Mam^ΔC^* mutant embryos also contained 2 MP1 neurons per segment, similar to wild type (Figure 8A–F; [6]), while *N^55e11^* mutant embryos had approximately 6 Odd positive MP1 neurons per segment (Table S1). Previous studies demonstrated that *Notch* mutants contain 2 additional Odd-positive MP2 cells per segment and this was also true in *mam^ΔC^* mutants (Figure 8E). These results indicate *mam^ΔC^* mutants resemble wild type embryos and differ from *Notch* mutants in the number of MP1 neurons that form during embryogenesis.

Next, we examined the MP3 lineage which is located just posterior to MP1s within each segment and normally divides asymmetrically to produce 1 H cell (Figure 8G) and 1 H cell sib neuron (Figure 8J) in wild type embryos [6]. In *mam^ΔC^* mutant embryos, the H cell sib was not detected as assessed by *CG13565* expression (Figure 8K), while approximately 6 H cells that expressed tyrosine hydroxylase (TH) were found in each segment (Table S1; Figure 8H). This was similar to *N^55e11^* mutant embryos in which the H cell sib was absent (Figure 3L) and the number of H cells in each segment increased to 10 (Table S1; Figure 8I). These results indicate both *mam^ΔC^* and *N^55e11^* have similar functions in the asymmetric cell division of the MP3 midline lineage and are needed for the formation of the H cell sib. Moreover, the zygotic *N^55e11^* mutation had a significantly larger effect on the number of H cells that formed compared to the *mam^ΔC^* mutation (P = 0.0001; Table S1).

**Figure 9:**
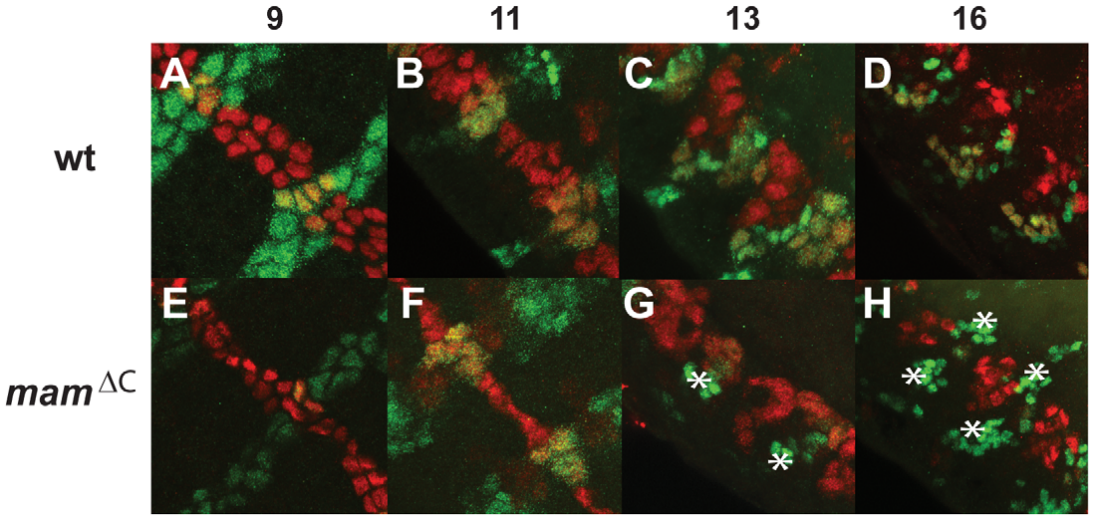
Midline cells that normally express *en* are absent in late *mam^ΔC^* mutant embryos. *En* (green) and *sim* (red) expression was monitored in (A–D) wild type and (E–H) *mam^ΔC^* mutant embryos during embryogenesis using specific antibodies. Prior to stage 10, 16 midline cells per segment are on the surface of the embryo (A and E), during stages 10 and 11, they delaminate into the developing nerve cord (B and F) and then differentiate into midline neurons and glia (D and H). En expression is indistinguishable in wild type (A and B) and *mam^ΔC^* mutant embryos (E and F) during stages 9–12, but diminishes in later stages in *mam^ΔC^* mutant embryos (G and H) compared to wild type (C and D). (A, B, E and F) Ventral or (C, D, G and H) lateral views of 2–3 segments are shown with anterior in the top, left-hand corner. Numbers above images indicate the developmental stage shown. At stage 16, iVUM neurons, the MNB and its progeny, and PMG express both *sim* and *en* and appear yellow, while the AMG and H cell express *sim* but not *en* and appear red. Asterisks indicate lateral CNS neurons that express *en*, but not *sim*.

Next, we examined lineages derived from MP4-6 found within the posterior of each segment. Each of these divide asymmetrically once to produce an iVUM and a mVUM, resulting in 3 of each per segment (see [6] and Table S1). The number of mVUMs increased from 3 cells per segment in wild type embryos (Figure 8M) to 11 in *mam^ΔC^* mutants as assessed with *Tyramine β hydroxylase* (*Tbh*), a specific marker for these midline cells (Table S1; Figure 8N). In *N^55e11^* mutant embryos, the number of mVUMs also increased to 11 per segment (Table S1; Figure 8O). To follow the iVUMs, which are also derived from MP4-6, as well as the MNB and its progeny, we assayed midline cells for the presence of En which is normally expressed in these midline neural lineages, as well as the PMG (see below). En was undetectable in the midline of *N^55e11^* mutants after stage 10 (data not shown), suggesting the iVUMs and the MNB and its progeny were absent. En protein levels appear relatively normal in *mam^ΔC^* mutants (Figure 9E and F) compared to wild type embryos (Figure 9A and B) until mid embryogenesis. During later developmental stages, each midline segment of wild type embryos contains 3 iVUMs and the progeny of the MNB, which divides multiple times after stage 11 to generate approximately 5–8 GABAergic neurons during embryogenesis [6]. However, only PMG express *en* in stage 13 *mam^ΔC^* mutants (Figure 9G) and eventually, these cells also disappear (Figure 9H; also see Figure 3), as they do in wild type embryos (Figure 9D). Moreover, all midline cells within *mam^ΔC^* mutant embryos remain at the dorsal side of the nerve cord (Figure 9G and H), which was also previously observed in *Notch^ts^* mutants [56]. The results suggest that *mam*, like *Notch*, is needed for the production of iVUMs during the asymmetrical cell divisions of MPs 4, 5 and 6 as well as for the development of the MNB and its progeny. In summary, midline neural phenotypes in *mam^ΔC^* mutant embryos are, in some cases, less severe, but consistent with midline phenotypes previously observed in *Dl^3^* mutants [6] and *N^55e11^* mutants (Table S1), with the exception of the MP1 neurons. The MP1 neurons appear unaffected in *mam^ΔC^* mutants, while *N^55e11^* mutants contain additional MP1s. Taken together, these studies of *mam^ΔC^* and *N^55e11^* mutants, together with previous experiments with *Dl^3^* mutants [6], indicate zygotic mutations in all 3 genes produce similar midline phenotypes of most neural subtypes. However, midline glia are eliminated and MP1 neurons expanded in *Notch* and *Dl* mutants, but not in *mam^ΔC^* mutants.

## Discussion

*Notch* has been shown to play multiple developmental roles in the CNS of several organisms [4], [7]–[9]. The *Drosophila* midline, with its easy to identify neural and glial lineages, has provided examples of multiple and reiterative roles of the *Notch* pathway within a single CNS lineage [6]. Here, the characterization of *mam^ΔC^* mutants indicates how a co-factor within a signaling pathway contributes to the development of different midline cell types and adds to our understanding of *Notch* signaling complexity.

**Figure 10:**
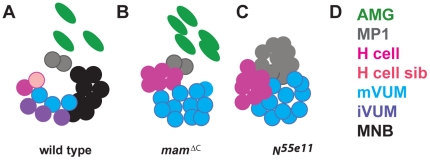
Comparision of CNS midline cell composition in wild type, *mam^ΔC^* and *N^55e11^* mutant embryos. *Drosophila* midline cells within a single segment at late embryogenesis (stage 16) are compared schematically in (A) wild type, (B) *mam^ΔC^* and (C) *N^55e11^* mutant embryos. (A) Wild type embryos contain 3 AMG and 6 different types of midline neurons. (B) *Mam^ΔC^* mutants contain 3 types of midline neurons and 5 AMG, whereas (C) *N^55e11^* mutant embryos contain 3 types of midline neurons and no AMG. (D) Each color corresponds to a particular midline cell type.

Initial activation of *sim* in the mesectoderm depends on maternal *Notch* expression [26], [56], [57], as *N^55e11^* germline clones lack most *sim* expression and therefore, contain few midline cells. Likewise, *mam^ΔC^* germline clones also show a reduction in *sim* expression. Thus, maternal contributions of both *mam* and *Notch* appear to act in the same pathway to activate *sim* early in development. Similarly, many midline neural phenotypes in zygotic *mam^ΔC^* mutant embryos are largely consistent with those of *N^55e11^* and *Dl^3^* [6], suggesting *mam* and *Notch* act together during the development of these neurons. *Notch* is required for formation of neurons expressing *en* [6] and may be needed to maintain *en* expression in midline cells that develop in the posterior compartment of each CNS segment, as first suggested by Bossing and Brand [27]. The results described here suggest *mam* is also required for the formation of the midline neurons that express *en* and develop into the iVUMs, the MNB and its progeny (Figure 10). While these cells of the posterior compartment were absent, the H cell and mVUM midline neurons were expanded in *mam^ΔC^* mutants (Figure 10), similar to *N^55e11^* and *Dl^3^* mutants, suggesting that *mam* function is needed within the *Notch* signaling pathway to obtain the variety of midline neurons found in wild type embryos [6].

The major difference we observed between zygotic *mam^ΔC^* and *N^55e11^* mutants was the presence of midline glia in *mam^ΔC^*, but not *N^55e11^* mutant embryos during mid to late embryogenesis (Figure 10). Not only were AMG present, but additional AMG survived in the mature CNS midline in *mam^ΔC^* mutants compared to wild type embryos (and *N^55e11^* mutants). The presence of AMG in *mam^ΔC^* mutants suggests either 1) the *mam^ΔC^* mutation is hypomorphic, 2) *mam* is not required within the *Notch* pathway for midline glial differentiation or 3) maternally deposited *mam* transcripts are stable and functional during the *Notch* signaling event needed for midline glial formation. Results with *mam* deficiency embryos indicated that midline glia formed and persisted in the complete absence of zygotic *mam* activity, suggesting it is not the hypomorphic nature of the *mam^ΔC^* allele that allows the midline glia to form. Currently, we cannot distinguish between the other two possibilities, although we favor the last hypothesis due to the timing of midline cell divisions. At gastrulation, each segment contains 8 mesectodermal cells, which each divide, resulting in 16 MPs per segment at stage 10. Cells that give rise to AMG and PMG do not divide again, whereas MPs that develop into neurons each divide once at stage 11. Because MPs that give rise to glia undergo their last division earlier than MPs that give rise to neurons, the *Notch* signaling event needed for midline glial differentiation may occur prior to *Notch* events that dictate midline neural fates at stage 11. Maternal Mam protein may linger just long enough to allow midline glia to form, but not long enough to function when MPs divide to give rise to midline neurons slightly later. We think this is the reason *N^55e11^* mutants contain more midline cells per segment than wild type (and *mam^ΔC^*; Table 2). In *N^55e11^* mutants, MPs that would normally form glia and not divide, instead take on neural fates and do divide. Our data are consistent with this hypothesis, but future, additional experiments are required to properly test it.

**Table 2:** Comparison of midline neurons and glia present within CNS segments of wild type, *N^55e11^* and *mam^ΔC^* mutant embryos during late embryogenesis.

aPrecursor cell	bCell type	cMarker	dSimExpression	eWild type	e *N^55e11^*	e *mam^ΔC^*
**MP1**	**MP1**	Odd	−	2	6	2
**MP3**	**H cell**	TH	−	1	10	6
**MP3**	**H cell sib**	CG13565	+	1	0	0
**MP4**	**mVUM**	Tbh	−	3	11	11
**MP4**	**iVUM**	En	+	3	0	0
**MP4**	**MNB and progeny**	En	+	5–8	0	0
**MP1 and 3**	**AMG**	Wrapper	+	3	0	5
**MP3 and 4**	**PMG**	Wrapper	+	0	0	0
	**Total**			18–21	27	24

a Midline precursors MP1, 3 and 4 are present during embryonic stage 10 give rise to the

b midline neural and glial subtypes listed in the second column [43].

c The various midline lineages were identified using the markers listed.

d All of the midline lineages that normally express *sim* in wild type embryos were absent in *N^55e11^* mutants, whereas all of the midline lineages that do not express *sim* in wild type embryos were present in *N^55e11^* mutants [29].

e The number of each cell type found in a single CNS segment of wild type, *N^55e11^* and *mam^ΔC^* mutant embryos at stage 16 is shown. The results obtained with *N^55e11^* mutant embryos were similar to those reported for *Dl^3^* mutants in a previous study [6].

In addition to this temporal sensitivity, *mam* may also be sensitive to spatially restricted events within the midline. Existing evidence suggests the 16 MPs fall into 3 equivalence groups at stage 10: the MP1s, MP3s and MP4s [6]. MP1s are in the anterior, MP3s in the middle and MP4s in the posterior of each CNS segment and effects of *mam^ΔC^* vary according to these positions. The results indicate that neurons derived from the anterior MP1s are sensitive to *N^55e11^*, but not *mam^ΔC^*; the middle MP3s are more sensitive to *N^55e11^* than *mam^ΔC^*; while the posterior MP4s are equally sensitive to *N^55e11^* and *mam^ΔC^*. In other words, *mam^ΔC^* mutants 1) differ with *N^55e11^* mutants in neurons derived from MP1s (MP1 neurons), 2) have similar, less severe effects compared to *N^55e11^* mutants in cells derived from the MP3s (the H cell and H cell sib) and 3) the same effects as *N^55e11^* mutants in cells derived from the posterior MP4s (mVUMs, iVUMS and MNB). These differences may be due to region specific differences in expression of other midline regulators that combine with Notch and/or Mam to control cell fate specification during embryogenesis [58]. Possible candidates include *hedgehog* and *wingless*, which are expressed in the midline, affect cell fate [27] and both interact with *mam* in a *Notch*-independent manner in other tissues [48], [59], [60]. In any case, clear differences in zygotic *mam* and *Notch* mutations within the midline exist and demonstrate that variations in different *Notch* signaling components can alter the cellular composition of the CNS in unique ways.

Close examination of *mam^ΔC^* and *N^55e11^* mutants during mid embryogenesis indicates they also differ in *sim* expression. After stage 10, *sim* diminishes in *N^55e11^* mutants, but persists in *mam^ΔC^* mutants. Likewise, midline glia, which are known to require *sim* expression to differentiate, do not develop in *N^55e11^* mutants, but do develop in *mam^ΔC^* mutants. Our data indicate that all midline lineages that normally express *sim* are absent in *N^55e11^* mutants, while midline lineages that do not normally express *sim* are present and expanded in zygotic mutants of *N^55e11^* (Table 2). Therefore, similar to the initiation of *sim* expression early, the maintenance of *sim* expression at this later time also appears to require zygotic *Notch* activity. In contrast, the results suggest *sim* expression persists in zygotic *mam^ΔC^* mutants.

In the canonical Notch pathway, Mam normally functions as a co-factor and collaborates with both the *NICD* and Su(H) to activate target genes. Consistent with this role, overexpression of *mam* alone does not affect the number of AMG generated at mid embryogenesis, whereas the overexpression of the *NICD* in wild type embryos increases AMG cell number [6]. Overexpression of the *NICD* in a *mam^ΔC^* mutant background still increased the number of AMG during this stage, further supporting the idea that zygotic *mam* is not needed at this time. During late embryogenesis, *mam^ΔC^* mutants contained extra AMG. Mutations in *mam* are known to promote neural tissue at the expense of ectoderm and this may result in the production of additional Spi, which inhibits apoptosis and allows extra midline glia to survive.

Altogether, the data suggest a high level of complexity in the regulation of CNS target genes of *Notch*. *Notch* likely interacts with additional cell-lineage specific co-activators other than, or in addition to, Mam in certain cells. In this way, combinatorial interactions between components of *Notch* signaling and other signaling pathways can lead to different outputs in various cell types, increasing cell diversity and function. The results described here indicate *mam^ΔC^* mutants contain AMG and PMG, whereas *N^55e11^* mutants do not. While this report describes major disruptions in *mam*, less severe mutations, such as small deletions, insertions or polymorphisms could also affect the midline and modify its cellular composition. Because *mam* mutations have more subtle effects on the midline compared to mutations in *Notch* or *Delta*, they may be tolerated more than mutations in major components of the pathway and actually contribute to CNS cellular variation in natural populations. Future experiments are needed to fully explore these functional differences between *mam* and *Notch* in the midline, as well as other tissues. Such differences can then be exploited to develop progressively specific research and clinical tools to regulate *Notch* signaling and the cellular composition of tissues [61], [62].

## Materials and Methods

### *Drosophila* strains

The *Drosophila* fly strain used in the genetic screen was homozygous for both the *UAS*-*GFP* and *sim*-*GAL4* transgenes which were recombined onto the same second chromosome. This combination labels all *Drosophila* midline cells beginning at developmental stage 10, through the remainder of embryogenesis and during larval stages. Prior to the mutagenesis screen, this line was isogenized using the *yw^67^* strain. The deficiency kit DK2, the 3 small deficiencies of *mam*: *Df(2R)BSC383*, *Df(2R)50C-38*, and *Df(2R)BSC18*, the *mam^8^* mutant line [34] and the *UAS-GFP* line were obtained from the Bloomington Stock Center. Additional fly lines used were: *N^55e11^* (described in [63]), *Dl^3^* [64], *mam^ΔC^* (this study), *sim*-*GAL4* [65], *UAS*-*NICD* and *UAS*-*Su(H)*.*VP16* [53], and *UAS*-*mam* [66]. The FLP–DFS technique was used to generate *mam^ΔC^* germline clones [54]. For this, the *mam^ΔC^* mutation was first recombined onto the FRT42B chromosome and then *w; P[48]42B 42B mam^ΔC^/CyO* virgins were crossed to *yw^67^ P{hs-FLP}; P{w^+^*, *FRT}42B*, *P{Ovo^D1^}55D/CyO* males. Next, 2–3 days old larvae with the genotype *y w P{hs-FLP}/w; P{w^+^*, *FRT}42B*, *P{Ovo^D1^}55D/P{w^+^*, *FRT}42B mam^ΔC^* generated from the cross were incubated at 37°C for 2 hours to induce recombination. Eclosed virgins were then crossed to *w; mam^ΔC^/CyO* males. Embryos collected from this cross were fixed and subjected to fluorescent *in situ* hybridization and immunohistochemistry. To test the effect of overexpressing the secreted form of Spi in *mam^ΔC^* mutants, the *mam^ΔC^* mutation was recombined onto both the *UAS-sspi4a* chromosome [67] and the *sim-GAL4* chromosome.

### Isolation of EMS generated *mam* mutants

To screen for genes on the second chromosome that affect midline development, *yw^67^*; *sim*-*GAL4 UAS*-*GFP* males were mutagenized with ethyl methylsulfonate (EMS) and then mated en mass to *yellow (y) white (w)^67^*; *Lobe (L)^2^*/*CyO Kruppel* (*Kr*)-*GFP* females. Single F1 male progeny were then backcrossed to 3 *yw^67^*; *L^2^*/*CyO Kr*-*GFP* virgin females in a single vial. Next, F2 siblings of the genotype *yw^67^*; *UAS-GFP sim-GAL4*/*CyO*, *Kr*-*GFP* were mated, and the absence of F3 progeny with straight wings indicated a line bearing a lethal second chromosome mutation (Figure 2A). To visually screen the lines bearing a lethal mutation on the second chromosome, embryos were collected every 12 hours, aged for 8 hours at room temperature and then examined for midline defects, first with a Leica MZ FLIII fluorescent stereomicroscope and then positives were more closely examined with a Zeiss Axioskop II fluorescent microscope and either a Zeiss Pascal or 710 confocal microscope. Homozygous mutant embryos were identified based on the absence of *Kr*-*GFP* fluorescence.

### DNA sequence analysis of the *mam^ΔC^* mutant

Genomic DNA was extracted from homozygous *mam^ΔC^* mutant embryos and used as a template to amplify all *mam* coding exons. After PCR amplification, each coding exon was cloned into the pSTblue-1 vector (Novagen) and then plasmids were sent to Alpha BioLab, Inc. for sequencing. Sequence analysis was performed using the FinchTV program (Geospiza, Inc.) and indicates the *mam^ΔC^* allele contains a point mutation that creates a premature stop codon. The resulting truncated protein ends at Mam residue 959, eliminating the C-terminal acid cluster and all 3 glycine-valine (GV) runs (Figure 2C). Based on comparison with *mam* deficiencies, the *mam^ΔC^* mutation behaves as a strong loss of function allele (Figure S2).

### Immunohistochemistry and *in situ* hybridization of embryos

Immunohistochemistry and *in situ* hybridization of whole mount embryos were performed as previously described [29], [68]. The following primary antibodies were used: mouse anti-β-galactosidase (1∶1000 Promega); rabbit anti-β-galactosidase (1∶2000 Cappel); rabbit anti-En (1∶100 Santa Cruz Biotech, Inc.); rat anti-Odd-skipped (1∶100), guinea pig anti-Odd-skipped (1∶100) and guinea pig anti-Runt (1∶100 or 1∶200 East Asian Distribution Center; EADC); rabbit anti-*GFP* (1∶500 Molecular Probes, Invitrogen); rat anti-Single-minded (1∶100 [69]; and rabbit anti-tyrosine hydroxylase (1∶500 [70]) and mouse anti-Wrapper (1∶5 Developmental Studies Hybridoma Bank). The anti-guinea pig Alexa 633 was used at 1∶100 and all other secondary antibodies were used at 1∶200: anti-rabbit Alexa 488, anti-guinea pig Alexa 488, anti-mouse Alexa 488, anti-rabbit Alexa 568, anti-rat Alexa 568, anti-mouse Alexa 568 (Molecular Probes, Invitrogen). Embryos were imaged with a Zeiss Pascal in the Forestry Department and Zeiss 710 laser scanning microscope in the Cellular and Molecular Imaging Facility at NCSU. To determine the number of cells belonging to each lineage, midline cells were labeled with specific markers and at least 8 thoracic segments within several embryos were counted and presented as the mean ± standard error of the mean (SEM) using stacked confocal images. The images shown are projections of multiple focal planes.

## Supporting Information

Figure S1 **Maternal** ***mam^ΔC^*** **mutations have more severe midline glial phenotypes than zygotic** ***mam^ΔC^*** **mutations.** Midline glial cells were labeled with a *wrapper* antibody (green; A–D, F, G and I) and either a *sim* antibody (red; A) or the BP102 monoclonal antibody (red; D, E, G and H). (A) *N^55e11^* mutant embryos do not express *wrapper*. The muscle phenotype characteristic of *Notch* mutants is indicated with the arrowhead. (B) Most embryos derived from *mam^ΔC^* germline clones did not express *wrapper*, although (C) low levels were detected in a few embryos. (G–I) Midline glia within *mam^ΔC^* mutant embryos contain extra processes that enwrap lateral axons compared to (D–F) wild type embryos. (D and G) The merge of *wrapper* and *BP102* is shown. (A) Ventral and (B–I) ventrolateral views of whole mount embryos are shown and anterior is toward the top, left corner.
(TIF)

Figure S2 **Unlike** ***N^55e11^*** **mutants, homozygous** ***mam*** **deficiency embryos contain AMG.** (A) A schematic map of regions uncovered by the *mam* deficiencies *Df(2R)BSC18*, *Df(2R)50C-38* and *Df(2R)BSC383* is shown. The top bar indicates the cytological bands that include the *mam* locus. *Mam* coding exons are indicated by green boxes and deletions are indicated with dotted lines. The entire *mam* locus is absent in deficiencies *Df(2R)BSC18* and *Df(2R)50C-38*, and the N-terminal region is absent in *Df(2R)BSC383*. This chromosomal region also contains several genes other than *mam* that are not shown. (B) In wild type embryos, Wrapper is expressed at a high level in the AMG (arrow) and at a low level in the PMG (arrowhead). (C–E) Wrapper expression was present in all three *mam* deletions. The midline glia in embryos homozygous for the deficiencies (D) *Df(2R)50C-38* and (E) *Df(2R)BSC383* appeared more disorganized than in embryos homozygous for deficiency (C) *Df(2R)BSC18*, which may be due to the absence of additional genes within these deletions. Whole mount embryos were labeled with an anti-Wrapper (red) antibody and ventral views of stage 13 embryos are shown; anterior is toward the top, left corner.
(TIF)

Table S1 **Comparison of MP1, H cell, mVUM midline neurons in wild type,** ***mam^ΔC^*** **and** ***N^55e11^*** **mutant embryos.**
(DOC)

Table S2 **Comparison of PMG in wild type and** ***mam^ΔC^*** **mutant embryos during mid and late embryogenesis.**
(DOC)
