# Supplementary figures and images for: Mastermind Mutations Generate a Unique Constellation of Midline Cells within the Drosophila CNS
Source: PLoS One. 2011 Oct 27;6(10):e26197. doi: 10.1371/journal.pone.0026197 (PMC3203113; doi:10.1371/journal.pone.0026197)

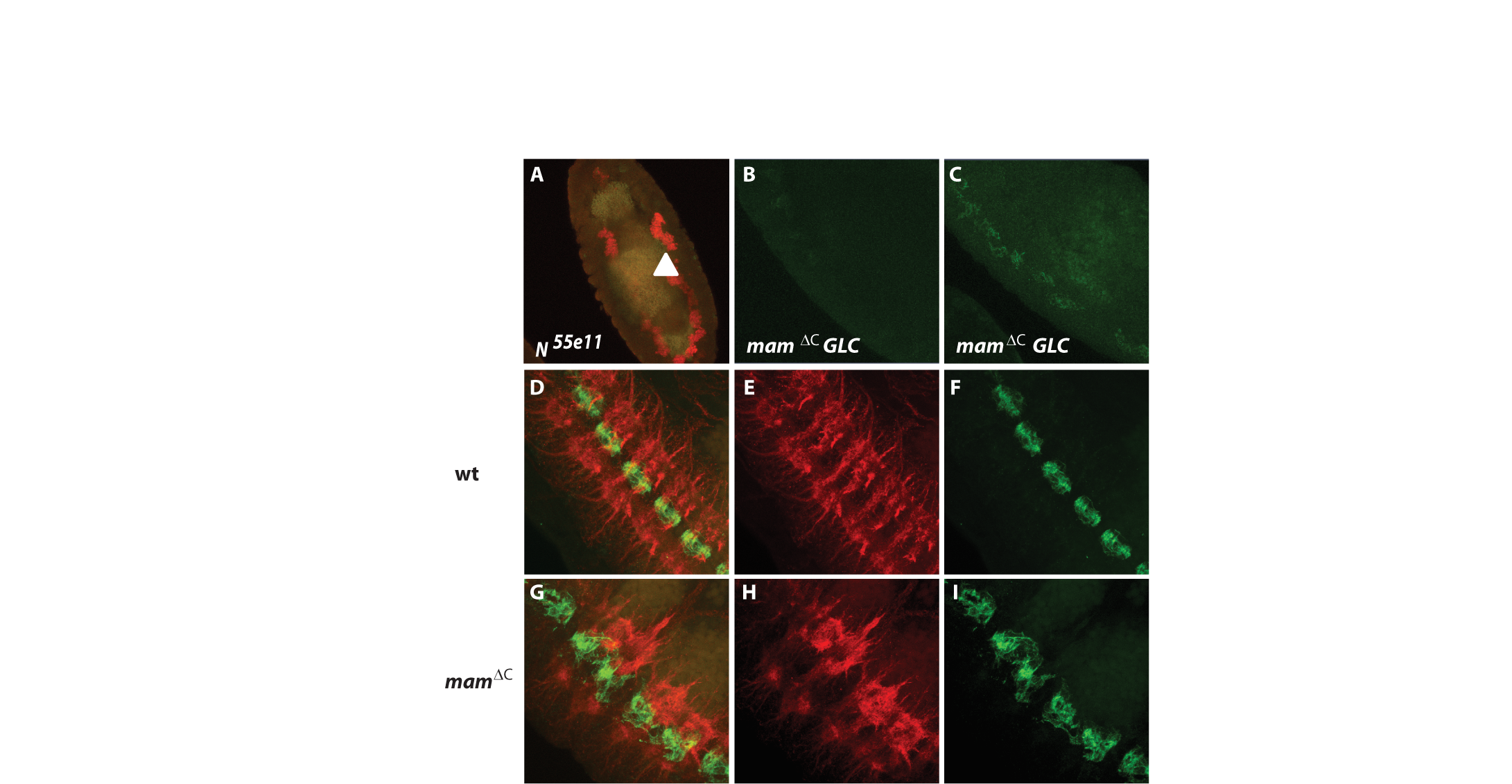

Supplement: Figure S1 — Maternal mamΔC mutations have more severe midline glial phenotypes than zygotic mamΔC mutations. Midline glial cells were labeled with a wrapper antibody (green; A–D, F, G and I) and either a sim antibody (red; A) or the BP102 monoclonal antibody (red; D, E, G and H). (A) N55e11 mutant embryos do not express wrapper. The muscle phenotype characteristic of Notch mutants is indicated with the arrowhead. (B) Most embryos derived from mamΔC germline clones did not express wrapper, although (C) low levels were detected in a few embryos. (G–I) Midline glia within mamΔC mutant embryos contain extra processes that enwrap lateral axons compared to (D–F) wild type embryos. (D and G) The merge of wrapper and BP102 is shown. (A) Ventral and (B–I) ventrolateral views of whole mount embryos are shown and anterior is toward the top, left corner. (TIF) [file pone.0026197.s001.tif]

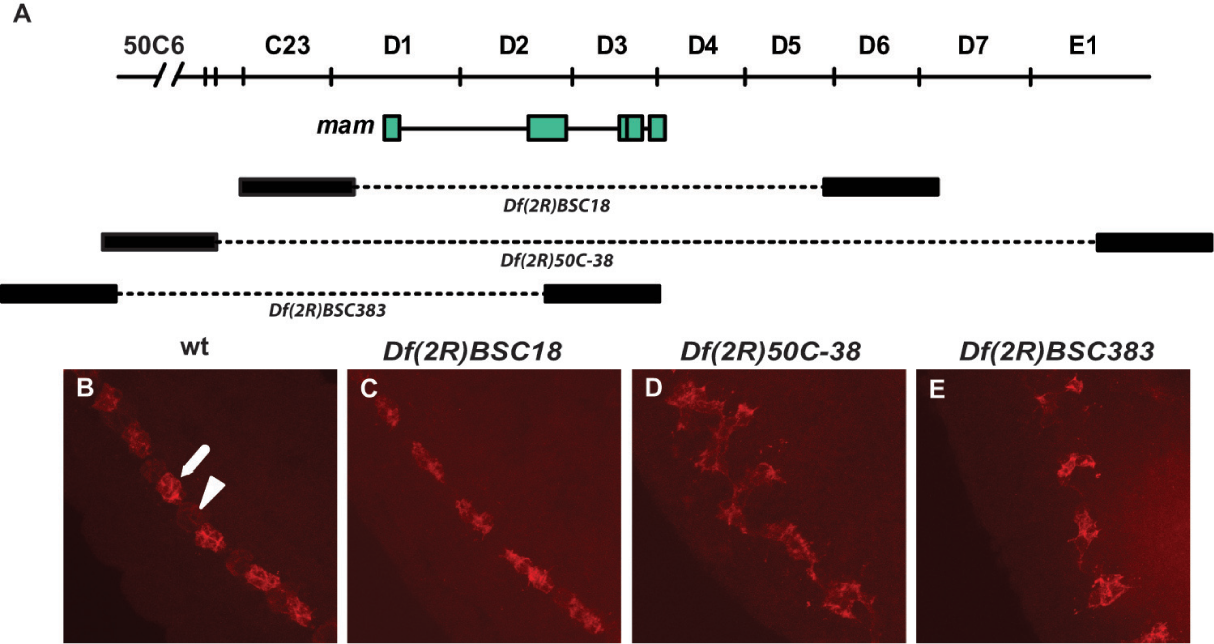

Supplement: Figure S2 — Unlike N55e11 mutants, homozygous mam deficiency embryos contain AMG. (A) A schematic map of regions uncovered by the mam deficiencies Df(2R)BSC18, Df(2R)50C-38 and Df(2R)BSC383 is shown. The top bar indicates the cytological bands that include the mam locus. Mam coding exons are indicated by green boxes and deletions are indicated with dotted lines. The entire mam locus is absent in deficiencies Df(2R)BSC18 and Df(2R)50C-38, and the N-terminal region is absent in Df(2R)BSC383. This chromosomal region also contains several genes other than mam that are not shown. (B) In wild type embryos, Wrapper is expressed at a high level in the AMG (arrow) and at a low level in the PMG (arrowhead). (C–E) Wrapper expression was present in all three mam deletions. The midline glia in embryos homozygous for the deficiencies (D) Df(2R)50C-38 and (E) Df(2R)BSC383 appeared more disorganized than in embryos homozygous for deficiency (C) Df(2R)BSC18, which may be due to the absence of additional genes within these deletions. Whole mount embryos were labeled with an anti-Wrapper (red) antibody and ventral views of stage 13 embryos are shown; anterior is toward the top, left corner. (TIF) [file pone.0026197.s002.tif]
