# Supplementary material for: Mastermind Mutations Generate a Unique Constellation of Midline Cells within the Drosophila CNS
Source: PLoS One. 2011 Oct 27;6(10):e26197. doi: 10.1371/journal.pone.0026197 (PMC3203113; doi:10.1371/journal.pone.0026197)
Supplement: Table S1 — Comparison of MP1, H cell, mVUM midline neurons in wild type, mamΔC and N55e11 mutant embryos. (DOC) [file pone.0026197.s003.doc]

***Table S1. Comparison of MP1, H cell, mVUM midline neurons in wild type, mam^ΔC^ and N^55e11^ mutant embryos.***

| **Genotype** | **MP1** | | | **H cell** | | | **mVUM** | | |
| --- | --- | --- | --- | --- | --- | --- | --- | --- | --- |
| **wild type** | 2.0 ± 0.00 | a | (16) | 1.1 ± 0.10 | a | (10) | 3.0 ± 0.00 | a | (12) |
| ***N^55e11^*** | 6.1 ± 0.42 | b | (13) | 9.6 ± 0.27 | b | (10) | 11.2 ± 0.28 | b | (19) |
| ***mam^ΔC^*** | 2.2 ± 0.11 | a | (22) | 5.6 ± 0.15 | c | (12) | 10.9 ± 0.25 | b | (19) |

The number of the indicated neurons found in a single CNS segment of wild type, *mam^ΔC^* and *N^55e11^*and at stage 16 is shown. Results are shown as means ± SEM and the sample size is indicated in parentheses.

MP1 ANOVA: *F*_2,48_=114.13, *P*=0.001, H cell ANOVA: *F*_2,29_=513.28, *P*=0.0001, and mVUM ANOVA: *F*_2,47_=291.11, *P*=0.0001. Within a column, treatments with different letters are significantly different (Tukey-Kramer HSD, *P*<0.05).
