# Supplementary material for: Mastermind Mutations Generate a Unique Constellation of Midline Cells within the Drosophila CNS
Source: PLoS One. 2011 Oct 27;6(10):e26197. doi: 10.1371/journal.pone.0026197 (PMC3203113; doi:10.1371/journal.pone.0026197)
Supplement: Table S2 — Comparison of PMG in wild type and mamΔC mutant embryos during mid and late embryogenesis. (DOC) [file pone.0026197.s004.doc]

***Table S2. Comparison of PMG in wild type and mam^ΔC^ mutant embryos during mid and late embryogenesis.***

| **Genotype** | **stage 13** | | | **stage 14** | | | **stage 15** | | | **stage 16** | | |
| --- | --- | --- | --- | --- | --- | --- | --- | --- | --- | --- | --- | --- |
| **wild type** | 4.1 ± 0.09 | a | (20) | 3.9 ± 0.10 | a | (17) | 2.1 ± 0.14 | b | (18) | 0.0 ± 0.00 | c | (24) |
| ***mam^ΔC^*** | 2.6 ± 0.50 | b | (10) | 2.6 ± 0.50 | b | (10) | 2.0 ± 0.24 | b | (9) | 0.09 ± 0.09 | c | (11) |

The number of PMG found in a single CNS segment of wild type and *mam^ΔC^* mutant embryos at stages 13, 14, 15 and 16 is shown. Results are shown as means ± SEM and the sample size is indicated in parentheses. ANOVA: *F*_7,111_=74.56, *P*=0.0001. Within the table, treatments with different letters are significantly different (Tukey-Kramer HSD, *P*<0.05).
